# A virtual reality paradigm simulating blood donation serves as a platform to test interventions to promote donation

**DOI:** 10.1038/s41598-024-60578-6

**Published:** 2024-05-06

**Authors:** Lisa A. Williams, Kallie Tzelios, Barbara Masser, Amanda Thijsen, Anne van Dongen, Tanya E. Davison

**Affiliations:** 1 School of Psychology, UNSW Sydney, Sydney, Australia; 2 School of Psychology, The University of Queensland, Brisbane, Australia; 3 Strategy and Growth, Australian Red Cross Lifeblood, Melbourne, Australia; 4 National Institute for Health and Care Research Blood and Transplant Research Unit in Donor Health and Behaviour, Department of Public Health and Primary Care, University of Cambridge, Cambridge, UK; 5 Department of Psychology, Health, and Technology, University of Twente, Enschede, The Netherlands; 6 Monash Art, Design and Architecture, Monash University, Melbourne, Australia; 7 Research and Innovation, Silverchain, Melbourne, Australia

**Keywords:** Human behaviour, Psychology

## Abstract

Effective interventions that support blood donor retention are needed. Yet, integrating an intervention into the time-pressed and operationally sensitive context of a blood donation center requires justification for disruptions to an optimized process. This research provides evidence that virtual reality (VR) paradigms can serve as a research environment in which interventions can be tested prior to being delivered in blood donation centers. Study 1 (N = 48) demonstrated that 360°-video VR blood donation environments elicit a similar profile of emotional experience to a live donor center. Presence and immersion were high, and cybersickness symptoms low. Study 2 (N = 134) was an experiment deploying the 360°-video VR environments to test the impact of an intervention on emotional experience and intentions to donate. Participants in the intervention condition who engaged in a suite of tasks drawn from the process model of emotion regulation (including attentional deployment, positive reappraisal, and response modulation) reported more positive emotion than participants in a control condition, which in turn increased intentions to donate blood. By showing the promise for benefitting donor experience via a relatively low-cost and low-resource methodology, this research supports the use of VR paradigms to trial interventions prior to deployment in operationally-context field settings.

## Introduction

Worldwide, since the advent of transfusion practices, medical procedures require access to a safe, sufficient, and stable blood supply^1^. Since 2015, the US whole-blood donor base has been in decline, with a similar pattern evidenced in US paid plasma collections since COVID-19. In the US, these declines have resulted in repeated emergency blood shortage callouts^2–4^, and worldwide shortages of plasma-derived medicines^5^. While blood product supply in Australia has been relatively stable^6,7^, demand for blood products has increased. In Australia, and worldwide, these pressures on supply are likely to intensify as populations age, and demand proportionally increases^8^. In many countries, the blood supply is reliant on volunteer blood donors^9^. Yet, relatively low proportions of the general population (0.6–5.3% in country-level analyses^9^) or the identified eligible population (14.2% in Australia^10^) donate blood. Further, despite return donors having a lower rate of blood-transmissible diseases than first-time donors^11^, 48–71% of donors do not return to donate again in the subsequent 5 years^12–14^.

In light of this, it is of critical importance to develop effective interventions that support blood donor retention^15^. However, blood donation centers are a complex setting. The workload demands on donation center staff are high, and blood collection agencies aim to minimize demands on donors’ time and maximize the quality of their experience. Integrating an intervention into this time-pressed and operationally sensitive context requires being able to justify disruptions to an optimized process^16^. The present research deployed a 360°-video virtual reality (VR) paradigm as a methodology to navigate the needs of research testing interventions to promote donor retention. The research first aimed to establish whether novel 360°-video VR blood donation center environments elicit a similar emotional response pattern to that observed in a live blood donation center (Study 1). The 360°-video VR blood donation center environments were then used as a platform to assess the potential efficacy of an intervention designed to optimally shape emotional experience (Study 2), providing the impetus for future deployment in centers.

A large body of research has revealed the psychological factors that motivate and deter blood donation (for reviews, see^15,17–19^), highlighting potential routes for intervention. Most of this research deploys a theoretical approach grounded in the theory of planned behavior^20^, identifying the role of attitudes, subjective norms, and perceived behavioral control in guiding intention to act, which in turn predicts action. Such approaches, however, often fail to capture the role of blood donors’ emotional experience in directing future donation behavior (cf.^21–23^), despite robust evidence pointing to the importance of emotions in guiding blood donation (for reviews, see^24,25^). Williams and colleagues^26^ argued for the importance of understanding the role of blood donor emotional experience for developing successful recruitment and retention campaigns.

Negative emotional experiences can be detrimental to donor recruitment and retention. General negative mood is associated with lower intentions to donate blood^27^. Turning to specific emotions, anticipated and experienced anxiety is negatively associated with intention to donate and donation behavior^22,28–30^. People who report more fear about donating report lower intentions to donate and are less likely to return to donate^29,31–35^. One exception to the general pattern of negative emotions undermining donor behavior is regret. Donors who anticipate experiencing regret if they did not donate in the future report stronger intentions to donate^23,30,33,36,37^.

Positive emotional experience is largely beneficial for donor recruitment and retention. Many donors report a positive emotional boost from donating^38^, often termed ‘warm-glow’^39,40^. Donors who report experiencing more warm glow report stronger intentions to donate^30,40^ and are more likely to return to donate again^41,42^; cf.^38^. Likewise, individuals who anticipate experiencing warm glow intend to, and indeed donate, more often^43^.

Focusing on donors’ emotional experience across the in-center donation process has provided key insights. The donation process from the waiting area (prior to blood draw) through to the refreshment area (following blood draw) is generally characterized by decreasing negative and increasing positive emotional experience^38,42,44^. However, not all donors’ emotional experience follows this general profile^45^—and donors’ temporal emotional profiles predict whether or not they return to donate. Specifically, donors who report a temporal emotion trajectory of medium–high/increasing joy, high calm, and low/decreasing stress across a donation are more likely to return to donate than those reporting a different emotional profile (e.g., high and decreasing stress)^46^.

Leveraging these findings on blood donors’ emotional experience to support donor recruitment and retention carries great promise^24,25,47^. This is particularly true for interventions that might be delivered in-center to promote positive and attenuate negative emotional states, given documented links between donors’ emotional states and future behavior. However, as mentioned above, deploying previously untested interventions live in donation centers is challenging given operational complexities.

Virtual reality (VR) is a research tool that can be of high utility in such circumstances. VR can effectively simulate a wide range of real-life contexts. To the degree that users subjectively experience “presence” in the virtual environment (i.e., “being there”)^48^, VR can elicit experiences in an immersive and poignantly realistic manner^49^. In addition, VR brings a number of benefits including isolation of target variables, consistency of experiential input across participants, and replication and sharing across research laboratories^50^.

A particular benefit of VR in research is resolving the identified tension between seeking ecological validity (best achieved in ‘real life’ settings) and maintaining experimental control (often achieved by utilizing laboratory settings)^51^. To this end, VR is especially useful in research areas where conducting field research is otherwise challenging^52^, such as consumer choice^53^, and risky behavior^54^. VR technology also has demonstrated utility in deploying psychological interventions, including those relating to vaccination^55–57^ and phobias and other mental health disorders^58–60^.

To date, the use of VR technology in blood donor psychology is limited. Two studies have utilized immersive VR technology to deliver interventions in blood donation centers. Bonk and colleagues tested the efficacy of audiovisual distraction delivered via a VR headset during a live blood draw in a donation center relative to business-as-usual procedures with no distraction^61^. Results indicated reduced self-reported vasovagal symptoms for participants in the VR distraction group who typically cope with stress by distracting themselves. Tarrant, Abrams, and Jackson tested the efficacy of an intervention delivered via a VR headset live in the waiting area of a blood donation center^62^. The intervention task guided participants to up-regulate their positive states across a 4.5-min session in which the visual display changed from bleak to lush. This VR-delivered intervention was effective in producing higher self-reported levels of calmness and happiness and lower levels of tension and fatigue relative to before the intervention. Neither study examined behavior-related outcomes in the form of donation intention, willingness to donate, or future behavior. Despite this limitation, such studies support the use of VR as an intervention tool in blood donation centers.

It is as yet unclear if VR can serve a different purpose: simulating the experience of being in a blood donation center. An experientially-valid virtual simulation would enable a range of research, including but not limited to assessing the potential efficacy of interventions to promote donor retention. Herein, we report two studies that sought to establish immersive 360°-video VR technology as a blood donor psychology research tool that can serve as the research environment for assessing intervention feasibility and efficacy. The aim of Study 1 was to test whether a novel set of two 360°-video VR environments depicting blood donation center waiting and refreshment areas elicit a similar profile of emotional experience to a live donor center^46^ and provide a high-quality immersive experience. Participants who had never before donated blood were instructed to imagine that they had arrived at a blood donation center to donate blood and then engaged with a 360°-video virtual blood donation center waiting area. After self-reporting their emotional experience and rating the qualities of the virtual experience, participants were instructed to imagine that they had completed the blood donation. Participants then engaged with a 360°-video virtual blood donation center refreshment area, after which they again self-reported their emotional experience and rated the qualities of the virtual experience. Participants’ levels of emotional experience were compared to levels reported by donors experiencing their first donation established in prior research^46^.

Study 2 constitutes an example of how VR paradigms can be used to establish the effectiveness of interventions prior to deploying them in complex real-life contexts. The aim of Study 2 was to show the promise of an intervention for benefitting blood donor experience and ultimately donor retention using the context of 360°-video VR environments. Specifically, Study 2 was an experiment testing the impact of an intervention on emotional experience and intentions to donate blood relative to a control condition. Participants randomly assigned to an intervention condition engaged in a suite of tasks drawn from the process model of emotion regulation^63^: attentional deployment (picturing something that elicits positive emotion), positive reappraisal (thinking about the positive impact of one’s behavior), and response modulation (a paced breathing task). These techniques are effective in producing lower levels of stress, and higher levels of positive emotions^64–66^, a profile of emotions that may promote intentions to donate blood, given that low stress paired with high positive emotion represents an optimal emotional profile for donor return^46^. In the control condition, participants were asked to complete a task designed to be similarly cognitively engaging, but emotionally-neutral in nature.

## Results

### Study 1

#### VR qualities, donation intention, and willingness to donate

Supporting the premise that the 360°-video virtual environments produced a high-fidelity experience, participants reported low levels of cybersickness symptoms, and high levels of presence and environment-specific immersion, after both the waiting area and refreshment area environments (see Table 1). All values deviated significantly from the midpoint of possible scores (2.5 for cybersickness; 4 for presence and immersion), *t*s > 8.77, *p*s < 0.001, *d*s > 1.27.

**Table 1:** Descriptive statistics for VR quality measures in Studies 1 and 2, and donation intention and willingness to donate in Study 1 by setting area.

	Waiting Area	Refreshment Area
	*M (SD)*	*M (SD)*
Study 1 (*N* = 48)		
Cybersickness symptoms	1.34 (0.32)	1.36 (0.36)
Presence	5.03 (0.68)	5.16 (0.91)
Environment-specific immersion	5.68 (0.99)	5.83 (1.01)
Donation intention	2.22 (1.43)	2.51 (1.47)
Willingness to donate	4.24 (1.86)	4.39 (1.96)
Study 2 Control Condition (*N* = 67)		
Cybersickness symptoms	1.49 (0.37)	1.42 (0.41)
Presence	4.56 (0.74)	4.59 (0.90)
Environment-specific immersion	5.13 (1.07)	5.27 (1.25)
Study 2 Intervention Condition (*N* = 67)		
Cybersickness symptoms	1.42 (0.38)	1.32 (0.34)
Presence	4.77 (0.98)	5.01 (0.93)
Environment-specific immersion	5.46 (1.08)	5.64 (1.02)

Possible ranges for these measures were 1–4 for cybersickness, and 1–7 for presence, donation intention, willingness to donate, and environment-specific immersion.

Donation intention levels were overall low-moderate and willingness to donate levels were moderate. Donation intention levels were significantly lower than midpoint of the scale (4), *t*s > 7.02, *p*s < 0.001, *d*s > 1.01. Levels of willingness to donate did not significantly differ from the midpoint of the scale (4), *t*s < 1.36, *p*s > 0.09, *d*s < 0.20.

#### Emotional experience

Levels of serene/content/peaceful and stressed/nervous/overwhelmed reported during the waiting area VR environment and serene/content/peaceful in the refreshment area VR environment did not significantly differ from average levels reported by first-time donors in these areas in a real donor center ^46^ (see Table 2). Equivalence tests^67^ assessing whether the mean difference between the Study 1 and in-center levels exceeded 0.5 points on the 5-point scale supported the absence of a difference of this magnitude (i.e., equivalence). Levels of joyful/glad/happy reported during the waiting area and refreshment area VR environments were significantly lower than average levels in-center, but still moderate in absolute terms. Equivalence tests supported the presence of differences exceeding 0.5 points on the 5-point scale in these cases (i.e., non-equivalence). Levels of stressed/nervous/overwhelmed in the refreshment area VR environment were significantly higher than average levels in-center, but still low in absolute terms and were equivalent when evaluated against a 0.5 point difference on the 5-point scale. Descriptive statistics for the full modified Differential Emotions Scale, completed after each VR environment, appear in Supplementary Materials Table S1.

**Table 2:** Descriptive statistics and comparisons to in-center levels for emotional experience in Study 1.

	Study 1 M* (SD)*	In-Center^46^ *M (SD)*	*t*	*p*	*d* [95%CI]	Equivalence tests (TOST) lower* t* (*p*)*;* upper* t* (*p*)
Waiting area						
Serene/content/peaceful	2.88 (0.88)	3.00 (1.06)	− 0.94	0.352	− 0.14 [− 0.42,0.15]	2.99 (0.002)*;* − 4.88 (< 0.001)
Joyful/glad/happy	2.40 (0.82)	3.24 (0.95)	− 7.15	< 0.001	− 1.03 [− 1.38,− 0.68]	− 2.87 (0.997); − 11.32 (< 0.001)
Stressed/nervous/overwhelmed	2.06 (0.88)	1.84 (0.95)	1.75	0.087	0.25 [− 0.04, 0.54]	5.67 (< 0.001); − 2.20 (0.016)
Refreshment area						
Serene/content/peaceful	3.24 (1.09)	3.25 (1.17)	− 0.03	0.974	− 0.01 [− 0.29,0.28]	3.11 (0.002); − 3.24 (0.001)
Joyful/glad/happy	2.74 (1.06)	3.48 (1.04)	− 4.82	< 0.001	− 0.70 [− 1.01, − 0.38]	− 1.57 (0.938); − 8.10 (< 0.001)
Stressed/nervous/overwhelmed	1.50 (0.83)	1.22 (0.56)	2.35	0.023	0.34 [0.05, 0.63]	6.51 (< 0.001); − 1.84 (0.036)

The possible range for each emotion measure was 1–5. Test statistics are one-sample *t-*tests of the distribution of data from Study 1 against the sample mean of emotion levels reported in-center ^46^, reported in the second column. TOST = two one-sided tests^67^.

### Study 2

#### VR qualities

As in Study 1, participants reported low levels of cybersickness symptoms, and high levels of presence and environment-specific immersion after both the waiting area and refreshment area environments (see Table 1). All values deviated significantly from the midpoint of possible scores (2.5 for cybersickness; 4 for presence and immersion), *t*s > 8.81, *p*s < 0.001, *d*s > 0.77.

Engaging with the emotional regulation intervention tasks did not significantly impact cybersickness nor environment-specific immersion relative to the control condition. Presence was higher in the intervention condition than the control condition in the refreshment area (*p* = 0.01), but not the waiting area (*p* = 0.18; interaction *F*(1,130) = 4.84, *p* = 0.03, ƞ_p_^2^ = 0.036). Equivalence tests^67^ assessing whether the mean difference between conditions exceeded 0.5 points on the 4-point scale for cybersickness or 1.0 points on the 7-point scale for presence and environment-specific immersion supported the absence of a difference of these magnitudes (i.e., equivalence; see Supplementary Materials Table S2). Full results of the general linear models on the VR quality variables are presented in Supplementary Materials Table S3.

Turning to task difficulty, overall absolute levels were low-to-moderate (see Table 3). The dot-counting task completed by participants in the control condition was rated as not statistically significantly different in difficulty as the response modulation (paced breathing) task in both the waiting and refreshment area environments. Equivalence tests^67^ assessing whether the mean difference between conditions exceeded 0.5 points on the 5-point scale supported the absence of a difference of this magnitude (i.e., equivalence). The other instructed tasks in the intervention condition (attentional deployment and positive reappraisal) were rated as significantly more difficult than the dot-counting task in the control condition; equivalence tests supported the presence of a difference exceeding 0.5 points on the 5-point scale (i.e., non-equivalence).

**Table 3:** Descriptive statistics and condition comparisons for task difficulty in Study 2.

	Control Condition *M (SD)*	Intervention Condition *M (SD)*	*t*	*p*	*d* [95%CI]	Equivalence tests (TOST) lower* t* (*p*)*;* upper* t* (*p*)
Waiting area						
Dot-counting	1.61 (0.80)					
Attentional deployment		2.14 (1.09)	3.16	.002	0.55 [0.21,0.90]	6.24 (< 0.001); 0.18 (0.572)
Positive reappraisal		2.12 (0.99)	3.27	0.001	0.57 [0.22,0.92]	6.50 (< 0.001); 0.06 (0.526)
Response modulation		1.54 (0.85)	− 0.51	0.609	− 0.09 [− 0.43,0.25]	3.02 (0.002); − 4.00 (< 0.001)
Refreshment area						
Dot-counting	1.49 (0.61)					
Positive reappraisal		2.00 (1.02)	3.48	< 0.001	0.60 [0.25,0.95]	6.96 (< 0.001); 0.07 (0.527)
Response modulation		1.59 (0.88)	0.75	0.455	0.13 [− 0.21,0.47]	4.59 (< 0.001); − 3.06 (0.001)

The possible range for these measures was 1–5, with higher numbers reflecting more ease. Test statistics in the fourth and fifth columns are independent-samples t-tests comparing rated difficulty of each of the intervention tasks to the rated difficulty of the control task. TOST = two one-sided tests^67^.

Examining relationships between task difficulty and presence in the intervention condition points to an inverse relationship between these constructs. Participants who found the response modulation and positive reappraisal tasks more difficult reported lower presence in both the waiting and refreshment area environments (*r*s <—0.28, *p*s < 0.025). Attentional deployment difficulty in the waiting area was not significantly associated with presence (*r* = 0.15, *p* = 0.228). In the control condition, dot-counting task difficulty and presence were not significantly associated (|*r|*s < 0.12, *p*s > 0.327).

#### Emotional experience and blood donation intention

Emotion experience varied as a function of intervention condition, *F*(1.65,237.5) = 4.66, *p* = 0.015, ƞ_p_^2^ = 0.034, Greenhouse-Geyser correction applied (see Table 4 for descriptive statistics and Supplementary Materials Table S4 for full model results). Comparison of estimated marginal means revealed that this interaction was driven by significantly higher levels of joyful/glad/happy in the intervention condition (*M* = 3.05) than in the control condition (*M* = 2.61, *p* = 0.001); serene/content/peaceful and stressed/nervous/afraid did not differ significantly by intervention condition (*p*s > 0.09). This interaction was not further qualified by waiting vs. refreshment area, *F*(1.80,237.5) = 1.54, *p* = 0.22, ƞ_p_^2^ = 0.012. Additional analyses exploring cybersickness symptoms, presence, environment-specific immersion, and task difficulty as potential moderators of the observed impact of condition on emotional states are reported in Supplementary Materials (Tables S5–S8). In brief, results suggest increased efficacy of the intervention tasks in producing the targeted emotional states when cybersickness symptoms were low, presence and immersion were high, and/or task difficulty was low. Descriptive statistics for the full modified Differential Emotions Scale, completed after each VR environment, appear in Supplementary Materials Table S9.

**Table 4:** Descriptive statistics for emotional experience, blood donation intention, and willingness to donate blood in Study 2.

	Control Condition *M (SD)*	Intervention Condition *M (SD)*
Waiting area		
Serene/content/peaceful	3.00 (0.97)	3.04 (1.13)
Joyful/glad/happy	2.54 (0.80)	2.84 (0.98)
Stressed/nervous/overwhelmed	1.97 (0.78)	1.88 (0.90)
Refreshment area		
Serene/content/peaceful	3.18 (0.85)	3.63 (0.98)
Joyful/glad/happy	2.69 (0.84)	3.25 (0.93)
Stressed/nervous/overwhelmed	1.49 (0.79)	1.40 (0.63)
Blood Donation Intention	2.46 (1.47)	2.63 (1.64)
Willingness to Donate Blood	4.96 (1.72)	5.31 (1.48)

The possible range for each emotion measure was 1–5. The possible range for blood donation intention and willingness to donate blood was 1–7.

**Figure 1:**
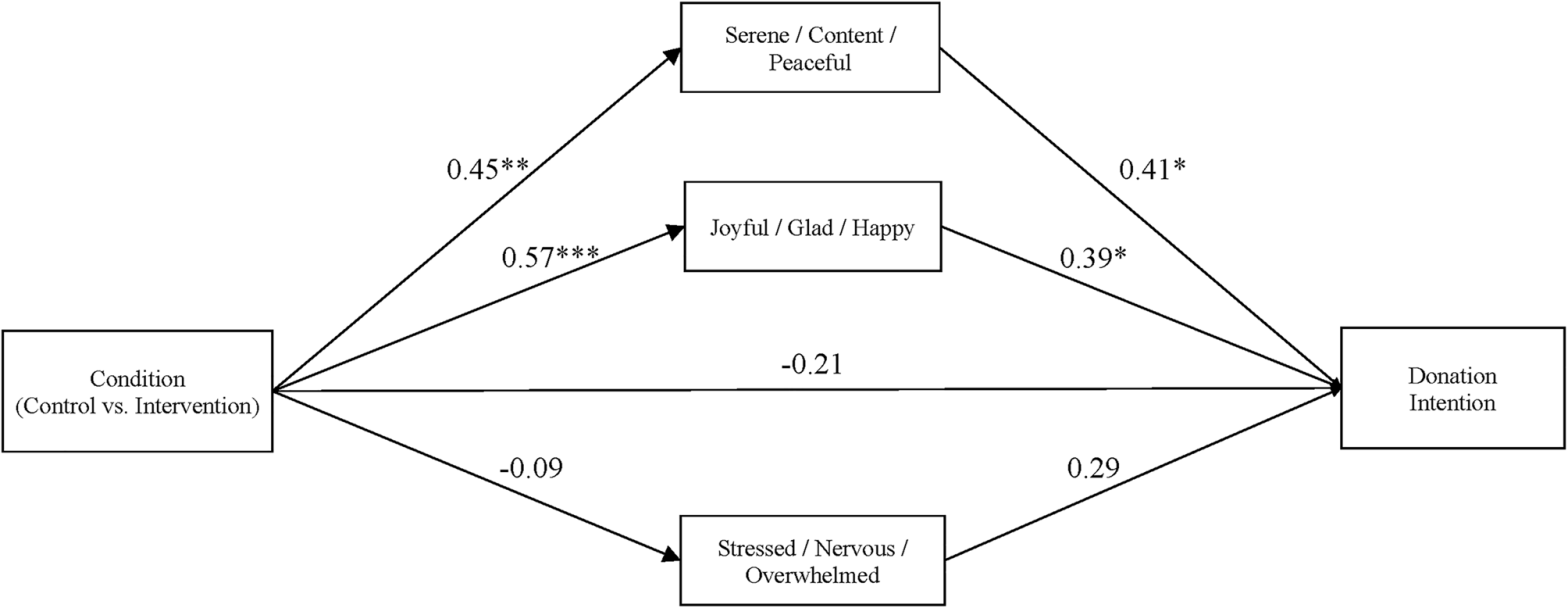
Path model estimating the relationship between intervention condition, emotions reported during the refreshment area environment, and intention to donate blood measured after the refreshment area environment in Study 2. Unstandardized estimates are reported. * *p* < .05, ** *p* < .01, *** *p* < .001.

Blood donation intention levels were relatively low, did not vary significantly according to condition, *t* (132) = 0.63, *p* = 0.53, *d* = 0.10 95%CI[− 0.23,0.45], and were equivalent across conditions relative to a difference of 1.0 point on the 7 point scale, TOST lower: *t* = 3.09, *p* = 0.001, TOST upper: *t* = − 4.35, *p* < 0.001 (see Table 4). However, the indirect effects of intervention condition on blood donation intention via both sets of positive states at the end of the refreshment area environment were nonzero (serene/content/peaceful: estimate = 0.376 95%CI[0.010, 0.445], joyful/glad/happy: estimate = 0.219 95%CI[0.033, 0.514]) (see Fig. 1). To the extent that engaging in the intervention tasks boosted levels of these positive states, intentions to donate blood were strengthened. The indirect effect via stressed/nervous/overwhelmed did not differ significantly from zero (estimate = − 0.026, 95%CI[− 0.143, 0.046]).

**Figure 2:**
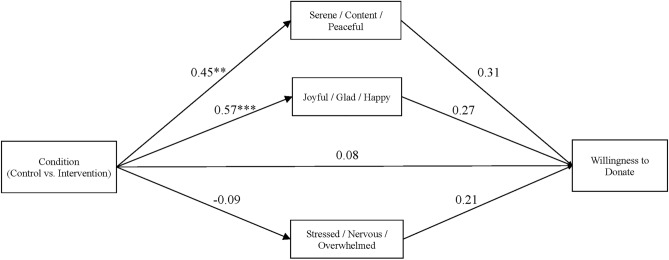
Path model estimating the relationship between intervention condition, emotions reported during the refreshment area environment, and willingness to donate blood measured after the refreshment area environment in Study 2. Unstandardized estimates are reported. ** *p* < 01, *** *p* < .001.

Donation willingness levels were relatively high, did not vary significantly according to condition, *t* (132) = 1.36, *p* = 0.21, *d* = 0.22 95%CI[− 0.12, 0.56], and were equivalent across conditions relative to a difference of 1.0 point on the 7 point scale, TOST lower: *t* = 2.34, *p* = 0.01, TOST upper: *t* = − 4.87, *p* < 0.001 (see Table 4). However, echoing a similar pattern observed for donation intention, the indirect effect of intervention condition on willingness to donate via serene/content/peaceful at the end of the refreshment area environment was nonzero (estimate = 0.138 95%CI[0.002, 0.372]) (see Fig. 2). To the extent that engaging in the intervention tasks boosted levels of serene/content/peaceful, willingness to donate blood was strengthened. The indirect effect via the other two emotions did not differ significantly from zero (joyful/glad/happy: estimate = 0.152 95%CI[− 0.044, 0.378], stressed/nervous/overwhelmed: estimate = − 0.019, 95%CI[− 0.113, 0.037]).

## Discussion

This research demonstrates the utility of immersive virtual reality (VR) as a research tool for blood donor psychology. Across two studies, the novel VR blood donation center environments utilizing 360° videos recorded in a real blood donation center produced a high-fidelity experience, indicated by high levels of presence and immersion, and low levels of cybersickness symptoms. Moreover, the 360°-video VR blood donation center environments served as a platform to assess the potential value of an intervention derived from emotion research for effective implementation in live donor centers.

Study 1 showed that the 360°-video virtual blood donation center waiting area and refreshment area environments elicited a similar profile of emotions as reported by donors live in-center^46^. Some emotion levels significantly differed between those observed in Study 1 and in-center, but the overall pattern of emotions seen in Study 1 pointed to one of relatively moderate positive emotion and low stress. Higher joyful/glad/happy levels in-center relative to in the 360°-video virtual environments in Study 1 may have arisen from having actually donated blood, an act with documented boosts to positive emotion^38,42,44^. Higher levels of stressed/nervous/overwhelmed in Study 1 relative to in-center may have arisen from several sources, including the novelty of using VR technology and the absence of interactions with center staff which reduce donors’ negative emotions^34^.

Study 2 demonstrated how VR paradigms can serve as a bridge to assess ease of use and efficacy of interventions prior to deployment in the operationally complex environment of a blood donation center. Broadly, the set of emotion regulation tasks deployed in the 360°-video VR waiting area and refreshment area environments was efficacious in promoting a profile of moderate positive emotion and low stress, a profile that in other research was associated with donor return^46^. Participants who engaged with the intervention tasks reported higher levels of joyful/glad/happy relative to those who engaged with the control tasks. Heightened levels joyful/glad/happy (and serene/content/peaceful) resulting from the intervention tasks had downstream effects on intentions to donate blood. Downstream effects on willingness to donate blood were carried via serene/content/peaceful. These results support the intervention’s possible efficacy in promoting future behavior if deployed in-center. These results also highlight the importance of considering positive emotions in the blood donation context. Negative emotion states such as anxiety, stress, fear, and disgust are frequently the focus of research into the psychological experience of blood donation^33,68–72^. Positive emotions, however, are frequently reported by blood donors, and are predictive of intentions to donate again and future behavior^26,46^. The present findings underscore the importance of considering both positive and negative states^73,74^—both to understand donors’ psychological experience and to best approach intervening to promote future donation behavior.

The intervention tasks neither enhanced nor undermined immersion or cybersickness. The findings on presence were nuanced. Overall, presence was higher in the refreshment area environment in the intervention condition than the control condition (but no different in the waiting area environment), suggesting that the intervention tasks may have increased presence. Within the intervention condition, higher perceived task difficulty was associated with lower presence, introducing the caveat that the intervention tasks may decrease presence to the degree that users found them difficult. Results of analyses of moderation patterns suggest increased efficacy of the intervention tasks in producing the targeted emotional states when cybersickness symptoms were low, presence and immersion were high, and/or task difficulty was low.

Broadly, this research demonstrates the utility of VR technology for donor psychology research. VR blood donation center settings can stimulate a similar psychological response as seen in live blood donor centers. Further, this research shows how such VR environments can be used to test interventions designed to promote donor retention. This evidence provides blood collection agencies with the justification required to introduce this or similar interventions into the operationally complex setting of a blood donation center. One benefit of having deployed the interventions via VR technology is that they are ready-made for implementation in-center via VR headsets^61,62^, or more simply via electronic tablet. Another potential use for the 360°-video VR paradigm developed in this work is to support donors who experienced an adverse event during their initial donation. Alongside catered communications^75^, engaging in a VR simulation of the blood donation experience, including a successful conclusion, may reduce the barriers to return among donors with a prior adverse event^76^.

Several limitations should be acknowledged. Our 360°-video virtual environments did not depict the blood draw. Simulating the blood draw process from a first-person vantage while representing the user’s body in a realistic way (matching body shape, skin color, and other visual cues) represents a logistical challenge. To our knowledge, only one prior study has utilized 360° videos to create a virtual reality simulation of a blood draw. Meindl and colleagues^77^ arranged the single participant’s father to record 360° video of himself undergoing a blood draw. The participant then engaged with the 360° video as part of an exposure therapy paradigm for needle fear. While innovative, this approach cannot be deployed at scale. One other study to date has used an immersive virtual reality blood draw as a stimulus. Jiang and colleagues^78^ presented participants with graded exposure to blood-injection-injury phobia stimuli in computer-generated immersive virtual environments, one of which included a blood draw in a clinical setting. Computer-generated VR environments carry the most promise for future research exploring psychological reactions to the blood draw (and how to intervene on them) in a blood donation context.

Our 360°-video VR environments were also limited in presenting the user’s view from a static vantage point. We were unable to replicate the experience of moving through the center, which may enhance immersion and presence^79^. Haptic feedback is another feature of VR environments that may enhance immersive experience^80^. We note that it is unlikely that such features, while desirable, would change the overall pattern of findings observed here in relation to the fidelity of the environments and the relative impact of the tested interventions on users’ psychological experience.

The set of emotion regulation tasks deployed in this research focused on three focal emotions identified in prior research as predictive of donor return in a study of states arising in-situ during the donation process: joyful/glad/happy, serene/content/peaceful, and stressed/nervous/overwhelmed^46^. Other emotions, however, are relevant in the blood donation context, including fear. Fear predicts adverse experiences during the donation process^31,81,82^ as well as future donation intention^33,81^ and behavior^31,83^. It will be important for future research to explore how VR technology might be used to examine and assess interventions to reduce fear and other related states among donors^34,84–86^.

In conclusion, this research supports the premise that VR is a useful research tool in the blood donation context, adding to the methodological toolkit of researchers working in this area. Advances in the use of VR in health psychology, neuroscience, and clinical medicine are well documented^51,52,87–90^. This research pushes the frontier of these advances to the field of donor psychology, showing how VR technology can simulate blood donation centers and provide a platform to assess interventions for donor retention.

## Method

### Virtual blood donation center environments

We created two virtual blood donation center environments for the purpose of this research: a waiting area and a refreshment area. These two areas were selected with advice from the Australian Red Cross Lifeblood, given positive inclinations to deliver interventions in these areas. We recorded 360° video using an Insta360 camera on a tripod in a real blood donation center in Sydney, Australia. Filming occurred outside of operating hours. We brought together a team of ‘actors’ to simulate other donors and the activities captured in the recordings were scripted to reflect ‘business-as-usual’ in a donor center.

Each environment was created using the A-frame platform (https://aframe.io/) by embedding the 360° video recordings into an interface that also provided instructions, faded-in and -out, and included additional audio tracks (e.g., medical machine beeping, background conversation) to enhance realism. All interactive elements of the program (e.g., self-reported emotional experience) were gaze-directed.

The web app was hosted locally on a Samsung S9 device using the Simple HTTP Server program (https://play.google.com/store/apps/details?id=jp.ubi.common.http.server). For data collection, the device was inserted into a Samsung VR headset. Data were recorded locally to the device.

The same virtual environments were used for Studies 1 and 2. The programming for the Study 2 environments included the control or intervention tasks, as detailed below.

### Study 1

#### Participants

Participants comprised a community sample of nondonors. We limited data collection to nondonors to replicate the sample from van Dongen et al.’s ^46^ analysis of emotion trajectories across first-time donors’ experience. Data from participants who had difficulty understanding instructions (*n* = 1), indicated not answering questions truthfully (*n* = 1), who self-reported being ineligible to donate blood (*n* = 11), or who had successfully donated or attempted to donate blood in the past (*n* = 22) were excluded from analysis. After exclusions, the total sample included 48 participants (62.5% female, *M*_age_ = 24.8, *SD*_age_ = 7.81). Most of the sample identified as South East Asian (45.8%), North East Asian (27.1%), South/Central Asian (12.5%) or White/Caucasian (10.4%). Half (50.0%) of the sample had never used a virtual reality headset and most participants reported no (27.1%) or very little (14.6%) expertise in virtual environments.

#### Procedure

Item wording and instructions are available in Supplementary Materials and on the OSF project page (https://osf.io/zfwpk/).

After providing informed consent, participants engaged in a brief demonstration VR environment, which served to familiarize participants with the headset and how to use eye gaze to respond to questions presented in the display. This environment included a static 360° image with embedded instructions.

Participants were then instructed to imagine that they had made an appointment to donate blood and that today was the day of the appointment. Participants were escorted to a different room, which included signage from a donation center. To enhance realism of the imagined scenario, participants were asked to imagine that they were in the waiting area of the donation center and that a receptionist asked them to complete a Donor Questionnaire. Participants completed a mock Donor Questionnaire adapted from that used by the local blood collection agency (see OSF project repository: https://osf.io/zfwpk/). Participants then put on the VR headset and engaged in the waiting area VR environment. Participants were encouraged to visually explore the environment while seated on a rotating chair.

Participants rated their current levels of joy, calm, and stress each minute for 4 min via eye gaze within the environment via eye-gaze. Three items from the modified Differential Emotions Scale (mDES)^91^, were selected based on their relevance to first-time blood donors’ return behavior ^46^: serene/content/peaceful (“How serene, content, or peaceful to you feel right now?”), joyful/glad/happy (“How joyful, glad, or happy do you feel right now?”), and stressed/nervous/overwhelmed (“How stressed, nervous, or overwhelmed do you feel right now?”). Ratings of each set of states within each VR environment were averaged across the four responses (one per minute for four minutes; calm: α_waiting area_ = 0.91, α_refreshment area_ = 0.95; joy: α_waiting area_ = 0.94, α_refreshment area_ = 0.95; stress: α_waiting area_ = 0.87, α_refreshment area_ = 0.93). Analysis of variation across the four timepoints is reported fully in the Supplementary Materials; negligible differences support the averaging approach deployed here.

Participants then removed the headset and completed several questionnaires on a desktop computer. These included the full 20-item mDES comprising 10 positively-valenced and 10 negatively-valenced items, measures of intention to donate blood (3 items^92^, α_waiting area_ = 0.95, α_refreshment area_ = 0.95) and willingness to donate blood (2 items, α_waiting area_ = 0.76, α_refreshment area_ = 0.82). Participants also reported the severity of experience of a selected set of six cybersickness symptoms drawn from the Simulator Sickness Questionnaire^93^. Responses were averaged to form an index of cybersickness symptoms (α_waiting area_ = 0.63, α_refreshment area_ = 0.70). Participants also completed the 15-item Brief Presence Questionnaire^94^. Overall presence was scored as the mean across items (α_waiting area_ = 0.78, α_refreshment area_ = 0.90). Participants also completed a novel 5-item measure assessing immersive qualities of the VR environments (e.g., “The virtual reality environment of the Blood Donation Center [waiting/refreshment] area seemed realistic to me;”; α_waiting area_ = 0.82, α_refreshment area_ = 0.84).

Participants were then instructed to imagine that they had now successfully given blood and that they had been escorted to the refreshment area. Participants then engaged with the refreshment area VR environment. Participants again rated their current levels of the three sets of emotions each minute for four minutes. Participants removed the headset and again completed the same series of questionnaires (including emotion, intention and willingness to donate blood, cybersickness, presence, and immersion) on a desktop computer. Participants were debriefed and thanked for their time.

#### Analytic approach

Data analyses were performed in IBM SPSS Statistics (v27). Descriptive statistics were computed for VR experience measures (simulator sickness, presence, environment-specific immersion) and for in-VR emotional states (serene/content/peaceful, joyful/glad/happy, stressed/nervous/overwhelmed). Levels of in-VR emotional states were compared against levels reported in-situ by donors in the waiting and refreshment area of a real donation center^46^. Equivalence tests were deployed using the two one-sided tests (TOST) approach^67^ to enable conclusions regarding the (non)equivalence between means observed in this study and in-center as observed in prior research ^46^. Due to space constraints, descriptive statistics of emotions rated after each VR environment are reported in Supplementary Materials Table S1.

### Study 2

#### Participants

Participants were undergraduate students who participated in exchange for partial course credit. Data from participants who had difficulty understanding (*n* = 1), experienced technical issues with the VR headset (*n* = 2), who were ineligible to donate blood (*n* = 17), and/or who had successfully donated or attempted to donate blood in the past (*n* = 29) were excluded from analysis. After exclusions, the total sample included 134 participants (61.9% female, *M*_age_ = 19.34, *SD*_age_ = 2.55). Most of the sample identified as North East Asian (35.8%), White/Caucasian (24.6%), South East Asian (23.1%) or South/Central Asian (10.4%). The majority of the sample (61.2%) had never used a virtual reality headset and most participants reported no (24.6%) or very little (13.4%) expertise in virtual environments.

#### Procedure

Barring the addition of the intervention or control tasks, the procedure for Study 2 mirrored that of Study 1. The environments programmed for Study 1 were adapted to accommodate the between-participants manipulation of intervention tasks, detailed in Table 3. Participants in the intervention condition were led through tasks that engaged attentional redeployment (picturing something that elicits positive emotion), positive reappraisal (thinking about the positive impact of donating blood), and response modulation (a controlled breathing task) during the waiting area environment. In the refreshment area environment, only the latter tasks were used. Participants in the control condition read instructions and engaged in a dot-counting task designed to be equally as engaging as the breathing animation task.

In Study 2, participants rated their levels of stress, joy, and calm once (rather than four times) in the VR-headset. After each VR environment, participants completed the full 20-item emotion measure, cybersickness (α_waiting area_ = 0.66, α_refreshment area_ = 0.75), presence (α_waiting area_ = 0.86, α_refreshment area_ = 0.89), and environment-specific immersion measures (α_waiting area_ = 0.85, α_refreshment area_ = 0.88) as per Study 1. Participants also rated the ease vs. difficulty of each task on 7-point scales. Ratings were reverse-scored so that higher scores indicated higher levels of task difficulty. Intention to donate (α_waiting area_ = 0.95) and willingness to donate (α = 0.82) were only measured after the refreshment area environment in Study 2.

#### Analytic approach

Data analyses were performed in IBM SPSS Statistics (v27). Mixed-effects general linear models assessed the impact of condition and VR environment on VR experience measures (cybersickness symptoms, presence, environment-specific immersion) separately. Independent-samples *t*-tests examining condition-wise differences were carried out on task difficulty, donation intention, and willingness to donate. Mixed-effects general linear models assessed the impact of condition (control vs. intervention, between-participants) and area (waiting vs. refreshment, within-participants) on in-VR emotions (joy, calm, stress; within-participants), and the role of cybersickness symptoms, presence, environment-specific immersion as potential moderators. Equivalence tests were deployed using the two one-sided tests (TOST) approach^67^ to enable conclusions regarding the (non)equivalence between means across conditions observed in this study. Due to space constraints, descriptive statistics for post-VR emotions are reported in Supplementary Materials Table S4.

Indirect effects analyses were performed using the PROCESS macro for SPSS^95^. The model included condition as the independent variable; in-VR refreshment area calm, joy, and stress as simultaneous mediators; and donation intention as the dependent variable. A separate model with the same predictors adopted willingness to donate as the dependent variable. Indirect effects were estimated via 10,000 bias-corrected bootstraps and evaluated via 95% confidence intervals.

### Ethical approval

This work was carried out with approval from the UNSW Human Research Ethics Advisory Panel (Approval #2931). All methods were performed in accordance with relevant guidelines and regulations. Informed consent was obtained from all participants.

### Supplementary Information

Supplementary Information.

## Data Availability

The datasets generated during and/or analyzed during the current study are available at the Open Science Framework: https://osf.io/zfwpk/.

## Code availability

The code used for data analysis for the current study is available at the Open Science Framework: https://osf.io/zfwpk/.

## References

[1] McCullough J, McCulllough J (2021). The blood supply. Transfusion Medicine.

[2] Young, M. US faces worst blood shortage in over a decade amid pandemic. *The Guardian* (2022).

[3] Red Cross facing a national blood shortage. *American Red Cross Blood Services* (2023).

[4] Mohtasham, D. Red Cross declares an emergency blood shortage, as number of donors hits 20-year low. *NPR* (2024).

[5] Plasma donations remain disappointingly low through ongoing pandemic, risking patients’ lives. *Plasma Protein Therapeutics Association* (2023).

[6] National Blood Authority Australia. *Annual Report 2022–2023*. https://blood.gov.au/sites/default/files/NBA0889%20%20NBA%202023%20Annual%20Report%20-%20WCAG%20Accessible.pdf (2023).

[7] National Blood Authority Australia. *Annual Report 2017–2018*. https://blood.gov.au/pubs/1718report/sites/default/files/publication/nba-annual-report-2017-18.pdf (2018).

[8] Fung YL (2022). The contribution and potential of older blood donors in Asia Pacific regions. Transfusion Med..

[9] World Health Organisation Health Product Policy and Standards Team. *Global Status Report on Blood Safety and Availability 2021*. https://www.who.int/publications/i/item/9789240051683 (2022).

[10] Mowat, Y. *et al.* Prevalence of blood donation eligibility in Australia: A population survey. *Transfusion (Paris)* (2023). 10.1111/trf.17474 PMC10952191 37464879

[11] Gemelli CN, Hayman J, Waller D (2017). Frequent whole blood donors: Understanding this population and predictors of lapse. Transfusion (Paris).

[12] Kheiri S, Alibeigi Z (2015). An analysis of first-time blood donors return behaviour using regression models. Transfusion Med..

[13] Kasraian L, Hosseini S, Dehbidi S, Ashkani-Esfahani S (2020). Return rate in blood donors: A 7-year follow up. Transfusion Med..

[14] Schreiber GB (2005). First year donation patterns predict long-term commitment for first-time donors. Vox Sang.

[15] Bagot KL, Murray AL, Masser BM (2016). How can we improve retention of the first-time donor? A systematic review of the current evidence. Transfus. Med. Rev..

[16] Masser B, Smith G, Williams LA (2014). Donor research in Australia: Challenges and promise. Transfus. Med. Hemother..

[17] Bednall TC, Bove LL, Cheetham A, Murray AL (2013). A systematic review and meta-analysis of antecedents of blood donation behavior and intentions. Soc. Sci. Med..

[18] Conner M, Armitage CJ (1998). Extending the Theory of Planned Behavior: A review and avenues for further research. J. Appl. Soc. Psychol..

[19] van Dongen A (2015). Easy come, easy go: Retention of blood donors. Transfus. Med..

[20] Ajzen I (1991). The theory of planned behavior. Organ. Behav. Hum. Decis. Process..

[21] Lemmens KPH (2009). Modelling antecedents of blood donation motivation among non-donors of varying age and education. Br. J. Psychol..

[22] Robinson NG, Masser BM, White KM, Hyde MK, Terry DJ (2008). Predicting intentions to donate blood among nondonors in Australia: An extended theory of planned behavior. Transfusion (Paris).

[23] Masser BM, White KM, Hyde MK, Terry DJ, Robinson NG (2009). Predicting blood donation intentions and behavior among Australian blood donors: Testing an extended theory of planned behavior model. Transfusion (Paris).

[24] Ferguson E, Masser B (2018). Emotions and prosociality: Lessons for blood donation. Aff. Determinants Health Behav..

[25] Masser B, Ferguson E, Merz EM, Williams L (2020). Beyond description: The predictive role of affect, memory, and context in the decision to donate or not donate blood. Transfus. Med. Hemother..

[26] Williams LA, Masser B, van Dongen A, Thijsen A, Davison T (2018). The emotional psychology of blood donors: A time-course approach. ISBT Sci. Ser..

[27] Sheldon KM (2022). Blood donation motivation in the United States and Russia: What keeps donors coming back?. J. Commun. Appl. Soc. Psychol..

[28] Clowes R, Masser BM (2012). Right here, right now: The impact of the blood donation context on anxiety, attitudes, subjective norms, self-efficacy, and intention to donate blood. Transfusion (Paris).

[29] France CR (2021). Fear is associated with attrition of first-time whole blood donors: A longitudinal examination of donor confidence and attitude as potential mediators. Transfusion (Paris).

[30] Martín-Santana JD, Reinares-Lara E, Romero-Domínguez L (2020). Modelling the role of anticipated emotions in blood donor behaviour: A cross-sectional study. J. Econ. Psychol..

[31] France CR (2014). Fear of blood draws, vasovagal reactions, and retention among high school donors. Transfusion (Paris).

[32] France CR, France JL, Himawan LK, Lux P, McCullough J (2021). Donation related fears predict vasovagal reactions and donor attrition among high school donors. Transfusion (Paris).

[33] Gilchrist PT, Masser BM, Horsley K, Ditto B (2019). Predicting blood donation intention: The importance of fear. Transfusion (Paris).

[34] Gilchrist PT, Thijsen A, Masser BM, France CR, Davison TE (2021). Improving the donation experience and reducing venipuncture pain by addressing fears among whole-blood and plasma donors. Transfusion (Paris).

[35] Li Z (2021). Blood donation fear, perceived rewards, self-efficacy, and Intention to return among whole blood donors in China: A social cognitive perspective. Front Psychol..

[36] Bagot KL, Masser BM, White KM (2015). Using an extended Theory of Planned Behavior to predict a change in the type of blood product donated. Ann. Behav. Med..

[37] Godin G, Conner M, Sheeran P, Bélanger-Gravel A, Germain M (2007). Determinants of repeated blood donation among new and experienced blood donors. Transfusion (Paris).

[38] Greffin K (2021). In the mood for a blood donation? Pilot study about momentary mood, satisfaction, and return behavior in deferred first-time donors. Transfus. Med. Hemother..

[39] Andreoni J (1990). Impure altruism and donations to public goods: A theory of warm-glow giving. Econ. J..

[40] Ferguson E, Atsma F, de Kort W, Veldhuizen I (2012). Exploring the pattern of blood donor beliefs in first-time, novice, and experienced donors: Differentiating reluctant altruism, pure altruism, impure altruism, and warm glow. Transfusion (Paris).

[41] Ferguson E, Taylor M, Keatley D, Flynn N, Lawrence C (2012). Blood donors’ helping behavior is driven by warm glow: More evidence for the blood donor benevolence hypothesis. Transfusion (Paris).

[42] Piliavin JA, Callero PL, Evans DE (1982). Addiction to altruism? Opponent-process theory and habitual blood donation. J. Pers. Soc. Psychol..

[43] Conner M, Godin G, Sheeran P, Germain M (2013). Some feelings are more important: Cognitive attitudes, affective attitudes, anticipated affect, and blood donation. Health Psychol..

[44] Zillmer E, Glidden R, Honaker L, Meyer J (1989). Mood states in the volunteer blood donor. Transfusion (Paris).

[45] Esefeld M, Sümnig A, Alpen U, Grabe HJ, Greinacher A (2022). A cross-sectional study of blood donors’ psychological characteristics over 8 weeks. Transfus. Med. Hemother..

[46] Van Dongen A (2021). The impact of temporal trajectories of emotional experience on blood donor return. Ann. Behav. Med..

[47] Ferguson E, France CR, Abraham C, Ditto B, Sheeran P (2007). Improving blood donor recruitment and retention: Integrating theoretical advances from social and behavioral science research agendas. Transfusion (Paris).

[48] Metzinger TK (2018). Why is virtual reality interesting for philosophers?. Front. Robot. AI.

[49] Sanchez-Vives MV, Slater M (2005). From presence to consciousness through virtual reality. Nat. Rev. Neurosci..

[50] McCall C, Blascovich J (2009). How, when, and why to use digital experimental virtual environments to study social behavior. Soc. Pers. Psychol. Compass.

[51] Parsons TD (2015). Virtual reality for enhanced ecological validity and experimental control in the clinical, affective and social neurosciences. Front Hum. Neurosci..

[52] Martingano AJ, Persky S (2021). Virtual reality expands the toolkit for conducting health psychology research. Soc. Pers. Psychol. Compass.

[53] Ung CY, Menozzi M, Hartmann C, Siegrist M (2018). Innovations in consumer research: The virtual food buffet. Food Qual. Prefer..

[54] de-Juan-Ripoll C (2018). Virtual reality as a new approach for risk taking assessment. Front. Psychol..

[55] Vandeweerdt C (2022). Virtual reality reduces COVID-19 vaccine hesitancy in the wild: A randomized trial. Sci. Rep..

[56] Mottelson A, Petersen GB, Lilija K, Makransky G (2021). Conducting unsupervised virtual reality user studies online. Front. Virtual Real..

[57] Nowak GJ (2020). Using immersive virtual reality to improve the beliefs and intentions of influenza vaccine avoidant 18-to-49-year-olds: Considerations, effects, and lessons learned. Vaccine.

[58] Freeman D (2018). Automated psychological therapy using immersive virtual reality for treatment of fear of heights: A single-blind, parallel-group, randomised controlled trial. Lancet Psychiatry.

[59] Freitas JRS (2021). Virtual reality exposure treatment in phobias: A systematic review. Psychiatric Q..

[60] Powers MB, Emmelkamp PMG (2008). Virtual reality exposure therapy for anxiety disorders: A meta-analysis. J. Anxiety Disord..

[61] Bonk VA, France CR, Taylor BK (2001). Distraction reduces self-reported physiological reactions to blood donation in novice donors with a blunting coping style. Psychosom. Med..

[62] Tarrant J (2019). The impact of virtual reality on mood states prior to blood donation. Schol. J. Psychol. Behav. Sci..

[63] English T, Eldesouky L, Gross JJ, John OP, Robins RW (2021). Emotion regulation: Basic processes and individual differences. Handbook of Personality: Theory and Research.

[64] Ockhuijsen H, Van Den Hoogen A, Eijkemans M, Macklon N, Boivin J (2014). Clarifying the benefits of the positive reappraisal coping intervention for women waiting for the outcome of IVF. Hum. Reprod..

[65] Rood L, Roelofs J, Bögels SM, Arntz A (2012). The effects of experimentally induced rumination, positive reappraisal, acceptance, and distancing when thinking about a stressful event on affect states in adolescents. J. Abnorm. Child Psychol..

[66] Zaccaro A (2018). How breath-control can change your life: A systematic review on psycho-physiological correlates of slow breathing. Front Hum. Neurosci..

[67] Lakens D, Scheel AM, Isager PM (2018). Equivalence testing for psychological research: A tutorial. Adv. Methods Pract. Psychol. Sci..

[68] Hoogerwerf M (2015). Factors associated with psychological and physiological stress reactions to blood donation: A systematic review of the literature. Blood Transfus..

[69] Chell K, Waller D, Masser B (2016). The Blood Donor Anxiety Scale: A six-item state anxiety measure based on the Spielberger State-Trait Anxiety Inventory. Transfusion (Paris).

[70] Chen L, Zhou Y, Zhang S, Xiao M (2024). How anxiety relates to blood donation intention of non-donors: The roles of moral disengagement and mindfulness. J. Soc. Psychol..

[71] Viar MA, Etzel EN, Ciesielski BG, Olatunji BO (2010). Disgust, anxiety, and vasovagal syncope sensations: A comparison of injection-fearful and nonfearful blood donors. J. Anxiety Disord..

[72] Olatunji BO, Etzel EN, Ciesielski BG (2010). Vasovagal syncope and blood donor return: Examination of the role of experience and affective expectancies. Behav. Mod..

[73] Thijsen A, Masser B, Davison TE, van Dongen A, Williams LA (2023). Beyond fear: A longitudinal investigation of emotions and risk of a vasovagal reaction in first-time whole-blood donors. Transfusion (Paris).

[74] Masser B, Ferguson E, Merz EM, Williams L (2020). Beyond description: The predictive role of affect, memory, and context in the decision to donate or not donate blood. Transf. Med. Hemother..

[75] Thijsen A, Gemelli CN, Davison TE, Masser B (2023). A randomized controlled trial of post-donation communication materials to increase donor return following a vasovagal reaction. Transfusion (Paris).

[76] Thijsen A, Gemelli CN, Davison TE, Masser B (2022). Using the Health Action Process Approach to predict blood donation intentions and return behavior following a vasovagal reaction for whole blood and plasma donors. Transfusion (Paris).

[77] Meindl JN, Saba S, Gray M, Stuebing L, Jarvis A (2019). Reducing blood draw phobia in an adult with autism spectrum disorder using low-cost virtual reality exposure therapy. J. Appl. Res. Intell. Disab..

[78] Jiang MYW, Upton E, Newby JM (2020). A randomised wait-list controlled pilot trial of one-session virtual reality exposure therapy for blood-injection-injury phobias. J. Affect. Disord..

[79] Lo SY, Lai CY (2023). Investigating how immersive virtual reality and active navigation mediate the experience of virtual concerts. Sci. Rep..

[80] Zhang Z (2023). Active mechanical haptics with high-fidelity perceptions for immersive virtual reality. Nat. Mach. Intell..

[81] France CR, France JL (2018). Fear of donation-related stimuli is reported across different levels of donation experience. Transfusion (Paris).

[82] Ditto B, Gilchrist PT, Holly CD (2012). Fear-related predictors of vasovagal symptoms during blood donation: It’s in the blood. J. Behav. Med..

[83] Zucoloto ML (2019). Factors that contribute to blood donation behavior among primary healthcare users: A structural approach. Transfus. Apheresis Sci..

[84] France CR, France JL, Kowalsky JM, Cornett TL (2010). Education in donation coping strategies encourages individuals to give blood: Further evaluation of a donor recruitment brochure. Transfusion (Paris).

[85] Masser B (2016). Improving first-time donor attendance rates through the use of enhanced donor preparation materials. Transfusion (Paris).

[86] Hanson SA, France CR (2009). Social support attenuates presyncopal reactions to blood donation. Transfusion (Paris).

[87] Bohil CJ, Alicea B, Biocca FA (2011). Virtual reality in neuroscience research and therapy. Nat. Rev. Neurosci..

[88] Bell IH, Nicholas J, Alvarez-Jimenez M, Thompson A, Valmaggia L (2020). Virtual reality as a clinical tool in mental health research and practice. Dialogues Clin. Neurosci..

[89] Torous J (2021). The growing field of digital psychiatry: Current evidence and the future of apps, social media, chatbots, and virtual reality. World Psychiatry.

[90] Li L (2017). Application of virtual reality technology in clinical medicine. Am. J. Transl. Res..

[91] Fredrickson BL, Tugade MM, Waugh CE, Larkin GR (2003). What good are positive emotions in crises? A prospective study of resilience and emotions following the terrorist attacks on the United States on September 11th, 2001. J. Pers. Soc. Psychol..

[92] France JL (2014). Development of common metrics for donation attitude, subjective norm, perceived behavioral control, and intention for the blood donation context. Transfusion (Paris).

[93] Kennedy RS, Lane NE, Berbaum KS, Lilienthal MG (1993). Simulator Sickness Questionnaire: An enhanced method for quantifying simulator sickness. Int. J. Aviat. Psychol..

[94] Witmer BG, Jerome CJ, Singer MJ (2005). The factor structure of the presence questionnaire. Presence Teleoperators Virtual Environ..

[95] Hayes AF (2022). Introduction to Mediation, Moderation, and Conditional Process Analysis: A Regression-Based Approach.

