# Supplementary material for: A virtual reality paradigm simulating blood donation serves as a platform to test interventions to promote donation
Source: Sci Rep. 2024 May 6;14:10334. doi: 10.1038/s41598-024-60578-6 (PMC11074277; doi:10.1038/s41598-024-60578-6)
Supplement: Supplementary file 1 — Supplementary Information. [file 41598_2024_60578_MOESM1_ESM.docx]

**Supplementary Materials**

Williams, Tzelios, Masser, Thijsen, van Dongen, Davison

‘A Virtual Reality Paradigm Simulating Blood Donor Emotional Experience Serves as a Platform to Test Interventions to Promote Donation’

These Supplementary Materials include:

- Table S1 – Descriptive statistics for modified Differential Emotions Scale (mDES) items rated post-VR in Study 1
- Table S2 – Results of equivalence tests for between-condition comparisons in Study 2
- Results of the General Linear Models on focal emotions by timepoint in Study 1 within the waiting area (Figure S1) and refreshment area (Figure S2) VR environments
- Table S3 – Model estimates for the General Linear Model on virtual reality quality measures in Study 2
- Table S4 – Model estimates for the General Linear Model on emotion ratings in Study 2
- Results of the General Linear Models exploring moderation of the condition by emotion interaction by cybersickness symptoms (Table S5), presence (Table S6), environment-specific immersion (Table S7), and task difficulty (Table S8)
- Table S9 – Descriptive statistics for modified Differential Emotions Scale (mDES) items and willingness to donate blood rated post-VR in Study 2, by condition
- Measures

*Table S1.* Descriptive statistics for modified Differential Emotions Scale (mDES) items rated post-VR in Study 1

|  | Waiting Area  *M* (*SD*) | Refreshment Area  *M* (*SD*) |
| --- | --- | --- |
| mDES – positive emotion items |  |  |
| Amused, fun-loving, silly | 1.98 (1.02) | 2.04 (1.03) |
| Awe, wonder, amazement | 2.56 (1.13) | 2.48 (1.15) |
| Grateful, appreciative, thankful | 2.46 (1.01) | 2.63 (1.14) |
| Hopeful, optimistic, encouraged | 2.69 (0.99) | 2.69 (1.10) |
| Inspired, uplifted, elevated | 2.40 (0.98) | 2.54 (1.22) |
| Interested, alert, curious | 3.27 (1.07) | 2.87 (1.12) |
| Joyful, glad, happy | 2.42 (0.90) | 2.69 (1.17) |
| Love, closeness, trust | 2.35 (1.10) | 2.31 (1.08) |
| Proud, confident, self-assured | 2.54 (0.92) | 2.60 (1.14) |
| Serene, content, peaceful | 3.10 (0.99) | 3.25 (1.00) |
| mDES – negative emotion items |  |  |
| Angry, irritated, annoyed | 1.19 (0.49) | 1.17 (0.63) |
| Ashamed, humiliated, disgraced | 1.08 (0.28) | 1.04 (0.20) |
| Contemptuous, scornful, disdainful | 1.17 (0.48) | 1.10 (0.37) |
| Disgust, distaste, revulsion | 1.15 (0.46) | 1.15 (0.55) |
| Embarrassed, self-conscious, blushing | 1.40 (0.57) | 1.33 (0.66) |
| Guilty, repentant, blameworthy | 1.17 (0.52) | 1.15 (0.55) |
| Hate, distrust, suspicion | 1.23 (0.59) | 1.15 (0.51) |
| Sad, downhearted, unhappy | 1.25 (0.60) | 1.21 (0.54) |
| Scared, fearful, afraid | 1.81 (1.00) | 1.29 (0.62) |
| Stressed, nervous, overwhelmed | 1.90 (1.02) | 1.38 (0.70) |

*Table S2.* Results of equivalence tests for cybersickness symptoms, presence, and environment-specific immersion in Study 1

|  | TOST (lower)  *t* (*p*) | TOST (upper)  *t* (*p*) |
| --- | --- | --- |
| Waiting area |  |  |
| Cybersickness symptoms | 8.79 (< .001) | -6.64 (< .001) |
| Presence | 5.27 (< .001) | -8.07 (< .001) |
| Environment-specific immersion | 3.61 (< .001) | -7.16 (< .001) |
| Refreshment area |  |  |
| Cybersickness symptoms | 9.22 (< .001) | -6.15 (< .001) |
| Presence | 3.67 (< .001) | -8.98 (< .001) |
| Environment-specific immersion | 3.20 (< .001) | -6.95 (< .001) |

*Note*. Equivalence bounds for the TOST procedure were ±0.5 for cybersickness and ±1.0 for presence and environment-specific immersion.

**Results of the General Linear Models on focal emotions by timepoint in Study 1**

To assess variation in focal emotion levels by timepoint across the VR environment timepoints in Study 1, two general linear models were estimated, one for the waiting area VR environment and the other for the refreshment area VR environment. For each, a within-participants factor reflecting emotion (stressed/nervous/overwhelmed, joyful/glad/happy, and serene/content/peaceful), a within-participants factor reflecting timepoint (time 1, time 2, time 3, time 4), and their interaction were modelled.

For the waiting area VR environment, the linear/linear interaction between emotion and timepoint was significant, *F*(3.65, 171.48) = 4.21, *p* = .004, ƞ_p_^2^ = .082, Greenhouse-Geyser correction applied (see Figure S1). Follow-up comparisons of estimated marginal means revealed the following significant comparisons: levels of serene/content/peaceful were higher at time 1 than time 2 (*p* = .008), time 1 than time 3 (*p* = .01), and time 1 than time 4 (*p* = .012). Levels of stressed/nervous/overwhelmed were lower at time 1 than time 4 (*p* = .022) and lower at time 2 than time 4 (*p* = .033). Levels of joyful/glad/happy were higher at time 1 than time 4 (*p* = .02) and higher at time 3 than time 4 (*p* = .018). All other comparisons were nonsignificant.

For the refreshment area VR environment, neither the effect of timepoint (*F*(2.58,162.12) = .168, *p* = .894, ƞ_p_^2^ = .004) nor the interaction between timepoint and emotion (*F*(3.45,162.12) = .841, *p* = .487, ƞ_p_^2^ = .018) were significant (see Figure S2).

*Figure S1.* Levels of focal emotions, by measurement timepoint, reported within the waiting area VR environment. Error bars represent ±1 standard error of the mean.

*Figure S2.* Levels of focal emotions, by measurement timepoint, reported within the refreshment area VR environment. Error bars represent ±1 standard error of the mean.

*Table S3.* Model estimates for the General Linear Model on virtual reality quality measures in Study 2

| Model Estimates | *F* | *p* | ƞ_p_^2^ |
| --- | --- | --- | --- |
| *Cybersickness symptoms* |  |  |  |
| Condition | 1.88 | .173 | .014 |
| Area | 13.29 | < .001 | .093 |
| Condition * Area | 0.31 | .576 | .002 |
| *Presence* |  |  |  |
| Condition | 4.47 | .037 | .033 |
| Area | 7.32 | .008 | .053 |
| Condition * Area | 4.84 | .030 | .036 |
| *Environment-specific Immersion* |  |  |  |
| Condition | 3.83 | .052 | .029 |
| Area | 6.44 | .012 | .047 |
| Condition * Area | 0.19 | .664 | .001 |

*Note.* Condition = intervention vs. control, Area = waiting area vs. refreshment area.

*Table S4.* Model estimates for the General Linear Model on emotion ratings in Study 2

| Model Estimates | *F* | *p* | ƞ_p_^2^ |
| --- | --- | --- | --- |
| Condition | 5.46 | .021 | .040 |
| Area | 1.97 | .163 | .015 |
| Emotion | 167.45 | < .001 | .559 |
| Condition * Area | 6.37 | .013 | .046 |
| Condition * Emotion | 4.66 | .015 | .034 |
| Area * Emotion | 32.29 | < .001 | .197 |
| Condition * Area * Emotion | 1.54 | .218 | .012 |

*Note.* Condition = intervention vs. control, Area = waiting area vs. refreshment area, Emotion = serene/content/peaceful vs. joyful/glad/happy vs. stressed/nervous/overwhelmed.

***Analyses exploring moderation of the interaction between condition and emotion***

Additional general linear models assessed potential moderation of the observed condition by emotion interaction in the focal models by four variables: cybersickness symptoms, presence, environment-specific immersion, and task difficulty. For parsimony each of the former three moderators was included as the average across the post-waiting area and post-refreshment area ratings (α_cybersickness symptoms_ = .83, α_presence_ = .92, α_environment-specific immersion_ = .92). For the latter, the average of the intervention task vs. control task difficulty ratings were utilized (α = .82).

In all four models, the three-way interactions between condition, emotion, and the moderator were either significant or marginally significant (see Tables S4-S7). Given the consistency of this pattern, all interactions were further explored via estimated marginal means of condition differences for each of the three emotions at three values of the moderator: the mean, the mean +1 standard deviation and the mean -1 standard deviation. At high levels of cybersickness symptoms and task difficulty, and low levels of presence and environment-specific immersion, there were no condition-wise differences in any of the three emotions. At average levels of all four moderators, levels of joyful/glad/happy were higher in the intervention condition than the control condition (*p*s < .008). At average levels task difficulty, levels of serene/content/peaceful were higher in the intervention condition than the control condition at average levels (*p* = .009). All other comparisons were not statistically significant at average moderator levels. At low levels of cybersickness symptoms and task difficulty, and high levels of presence and environment-specific immersion, levels of joyful/glad/happy were higher in the intervention condition than the control condition (*p*s < .04). Further, levels of stressed/nervous/overwhelmed were lower in the intervention condition than the control condition at high levels of presence and environment-specific immersion (*p*s < .02), and levels of serene/content/peaceful were higher in the intervention condition than the control condition at high levels of environment-specific immersion and low levels of task difficulty (*p*s < .03).

Overall, these patterns broadly reflect a pattern of higher efficacy of the intervention tasks in shaping emotional states when participants were not experiencing cybersickness symptoms, were experiencing high presence and immersion, and/or when the tasks were not difficult. These findings should be interpreted with caution due to low evidentiary weight (due to marginal significance of some three-way interactions), but the consistency of patterns, especially for joyful/glad/happy, lend credence to a general effect worthy of replication attempts in future research.

*Table S5.* Model estimates for the General Linear Model on emotion ratings including cybersickness symptoms as a moderator in Study 2

| Model Estimates | *F* | *p* | ƞ_p_^2^ |
| --- | --- | --- | --- |
| Condition | 0.65 | .423 | .005 |
| Area | 0.65 | .421 | .005 |
| Emotion | 38.39 | < .001 | .231 |
| Cybersickness Symptoms | 0.15 | .695 | .001 |
| Condition * Area | 1.49 | .225 | .011 |
| Condition * Emotion | 3.89 | .028 | .030 |
| Condition * Cybersickness | 0.07 | .788 | .001 |
| Area * Emotion | 3.67 | .032 | .028 |
| Area * Cybersickness | 0.28 | .600 | .002 |
| Emotion * Cybersickness | 10.20 | < .001 | .074 |
| Condition * Area * Emotion | 0.43 | .628 | .003 |
| Condition * Area * Cybersickness | 0.46 | .500 | .004 |
| Condition * Emotion * Cybersickness | 2.73 | .076 | .021 |
| Area * Emotion * Cybersickness | 0.97 | .372 | .008 |
| Condition * Area * Emotion * Cybersickness | 0.90 | .400 | .007 |

*Table S6.* Model estimates for the General Linear Model on emotion ratings including presence as a moderator in Study 2

| Model Estimates | *F* | *p* | ƞ_p_^2^ |
| --- | --- | --- | --- |
| Condition | 0.66 | .419 | .005 |
| Area | 0.13 | .721 | .001 |
| Emotion | 0.75 | .387 | .006 |
| Presence | 19.42 | < .001 | .132 |
| Condition * Area | 1.49 | .225 | .011 |
| Condition * Emotion | 2.13 | .129 | .016 |
| Condition * Presence | 0.30 | .587 | .002 |
| Area * Emotion | 1.74 | .180 | .013 |
| Area * Presence | 0.01 | .908 | < .001 |
| Emotion * Presence | 5.26 | .009 | .039 |
| Condition * Area * Emotion | 0.12 | .866 | .001 |
| Condition * Area * Presence | 0.20 | .654 | .002 |
| Condition * Emotion * Presence | 2.93 | .064 | .022 |
| Area * Emotion * Presence | 5.24 | .008 | .039 |
| Condition * Area * Emotion * Presence | 0.72 | .916 | .001 |

*Table S7.* Model estimates for the General Linear Model on emotion ratings including environment-specific immersion as a moderator in Study 2

| Model Estimates | *F* | *p* | ƞ_p_^2^ |
| --- | --- | --- | --- |
| Condition | 0.01 | .922 | < .001 |
| Area | 0.03 | .876 | < .001 |
| Emotion | 0.14 | .840 | .001 |
| Environment-specific Immersion | 10.74 | < .001 | .007 |
| Condition * Area | 0.24 | .624 | .002 |
| Condition * Emotion | 5.93 | .005 | .044 |
| Condition * Immersion | 0.21 | .651 | .002 |
| Area * Emotion | 1.50 | .227 | .011 |
| Area * Immersion | 0.01 | .928 | < .001 |
| Emotion * Immersion | 7.07 | .002 | .052 |
| Condition * Area * Emotion | 0.01 | .985 | < .001 |
| Condition * Area * Immersion | 0.002 | .965 | < .001 |
| Condition * Emotion * Immersion | 7.55 | .001 | .055 |
| Area * Emotion * Immersion | 5.35 | .007 | .040 |
| Condition * Area * Emotion * Immersion | 0.07 | .924 | .001 |

*Table S8.* Model estimates for the General Linear Model on emotion ratings including task difficulty as a moderator in Study 2

| Model Estimates | *F* | *p* | ƞ_p_^2^ |
| --- | --- | --- | --- |
| Condition | 11.27 | .001 | .080 |
| Area | 0.81 | .371 | .006 |
| Emotion | 80.03 | < .001 | .383 |
| Task Difficulty | 5.32 | .023 | .040 |
| Condition * Area | 3.31 | .071 | .025 |
| Condition * Emotion | 7.62 | .001 | .056 |
| Condition * Difficulty | 6.18 | .014 | .046 |
| Area * Emotion | 0.27 | .744 | .002 |
| Area * Difficulty | 2.45 | .120 | .019 |
| Emotion * Difficulty | 18.19 | < .001 | .124 |
| Condition * Area * Emotion | 0.27 | .738 | .002 |
| Condition * Area * Difficulty | 1.36 | .246 | .010 |
| Condition * Emotion * Difficulty | 3.09 | .054 | .023 |
| Area * Emotion * Difficulty | 2.71 | .074 | .021 |
| Condition * Area * Emotion * Difficulty | 0.07 | .920 | .001 |

*Table S9.* Descriptive statistics for modified Differential Emotions Scale (mDES) items and willingness to donate blood rated post-VR in Study 2, by condition

|  | Control | |  | Intervention | |
| --- | --- | --- | --- | --- | --- |
|  | Waiting Area M (SD) | Refreshment Area M (SD) |  | Waiting Area M (SD) | Refreshment Area M (SD) |
| mDES – positive emotion items |  |  |  |  |  |
| Amused, fun-loving, silly | 2.03 (1.06) | 1.88 (0.98) |  | 1.82 (0.93) | 1.86 (1.06) |
| Awe, wonder, amazement | 2.43 (1.26) | 2.39 (1.18) |  | 2.35 (1.19) | 2.54 (1.24) |
| Grateful, appreciative, thankful | 2.52 (1.04) | 2.64 (1.18) |  | 2.69 (1.13) | 3.05 (1.14) |
| Hopeful, optimistic, encouraged | 2.42 (0.99) | 2.61 (1.11) |  | 2.65 (1.02) | 3.05 (1.02) |
| Inspired, uplifted, elevated | 2.13 (1.01) | 2.37 (1.11) |  | 2.43 (1.05) | 2.83 (1.11) |
| Interested, alert, curious | 3.27 (0.86) | 2.76 (1.09) |  | 3.31 (1.07) | 3.02 (1.11) |
| Joyful, glad, happy | 2.52 (0.89) | 2.66 (0.86) |  | 2.77 (1.06) | 3.14 (1.07) |
| Love, closeness, trust | 2.15 (1.00) | 2.19 (1.10) |  | 2.28 (1.04) | 2.42 (1.04) |
| Proud, confident, self-assured | 2.28 (0.92) | 2.40 (1.02) |  | 2.35 (1.01) | 2.95 (1.11) |
| Serene, content, peaceful | 2.96 (0.96) | 2.87 (0.89) |  | 3.11 (1.06) | 3.65 (0.96) |
| mDES – negative emotion items |  |  |  |  |  |
| Angry, irritated, annoyed | 1.13 (0.34) | 1.12 (0.37) |  | 1.12 (0.42) | 1.05 (0.21) |
| Ashamed, humiliated, disgraced | 1.09 (0.34) | 1.07 (0.32) |  | 1.06 (0.30) | 1.08 (0.37) |
| Contemptuous, scornful, disdainful | 1.21 (0.48) | 1.10 (0.39) |  | 1.11 (0.40) | 1.03 (0.17) |
| Disgust, distaste, revulsion | 1.09 (0.29) | 1.04 (0.21) |  | 1.09 (0.34) | 1.00 (0.00) |
| Embarrassed, self-conscious, blushing | 1.57 (0.74) | 1.21 (0.41) |  | 1.60 (0.88) | 1.14 (0.39) |
| Guilty, repentant, blameworthy | 1.09 (0.29) | 1.03 (0.17) |  | 1.15 (0.44) | 1.12 (0.42) |
| Hate, distrust, suspicion | 1.10 (0.31) | 1.06 (0.24) |  | 1.15 (0.40) | 1.03 (0.17) |
| Sad, downhearted, unhappy | 1.27 (0.48) | 1.16 (0.37) |  | 1.29 (0.66) | 1.14 (0.43) |
| Scared, fearful, afraid | 1.43 (0.76) | 1.19 (0.61) |  | 1.40 (0.73) | 1.14 (0.35) |
| Stressed, nervous, overwhelmed | 1.76 (0.85) | 1.57 (0.86) |  | 1.86 (0.90) | 1.37 (0.55) |

***Measures***

Emotion measures were adapted from the modified Differential Emotion Scale (Fredrickson et al., 2003).

**In-VR Emotions (adapted/selected from the modified Differential Emotion Scale [mDES]; Fredrickson et al., 2003)**

| **Instructions** | N/a |
| --- | --- |
| **Items** | 1. How serene, content, or peaceful do you feel right now? |
|  | 1. How joyful, glad, or happy do you feel right now? |
|  | 1. How stressed, nervous, or overwhelmed do you feel right now? |
| **Response options** | 5-point scales: *Not at all – A little bit – Moderately – Quite a bit – Extremely* |

**Post-VR Emotions – retrospective (adapted/selected from the mDES; Fredrickson et al., 2003)**

***Study 2 only***

| **Instructions** | **The next three questions are about your feelings while you were in Virtual Reality waiting/refreshment area.** |
| --- | --- |
| **Items** | 1. How serene, content, or peaceful did you feel in the Virtual Reality waiting/refreshment area? |
|  | 1. How joyful, glad, or happy did you feel in the Virtual Reality waiting/refreshment area? |
|  | 1. How stressed, nervous, or overwhelmed did you feel in the Virtual Reality waiting/refreshment area? |
| **Response options** | 5-point scales: *Not at all – A little bit – Moderately – Quite a bit – Extremely* |

**Post-VR Emotions – current (adapted/selected from the mDES; Fredrickson et al., 2003)**

| **Instructions** | The next set of questions asks about your current experience. |
| --- | --- |
| **Items** | 1. How amused, fun-loving, or silly do you feel right now? |
|  | 1. How angry, irritated, or annoyed do you feel right now? |
|  | 1. How ashamed, humiliated, or disgraced do you feel right now? |
|  | 1. How much awe, wonder, or amazement do you feel right now? |
|  | 1. How contemptuous, scornful, or disdainful do you feel right now? |
|  | 1. How much disgust, distaste, or revulsion do you feel right now? |
|  | 1. How embarrassed, self-conscious, or blushing do you feel right now? |
|  | 1. How grateful, appreciative, or thankful do you feel right now? |
|  | 1. How guilty, repentant, or blameworthy do you feel right now? |
|  | 1. How much hate, distrust, or suspicion do you feel right now? |
|  | 1. How hopeful, optimistic, or encouraged do you feel right now? |
|  | 1. How inspired, uplifted, or elevated do you feel right now? |
|  | 1. How interested, alert, or curious do you feel right now? |
|  | 1. How joyful, glad, or happy do you feel right now? |
|  | 1. How much love, closeness, or trust do you feel right now? |
|  | 1. How proud, confident, or self-assured do you feel right now? |
|  | 1. How sad, downhearted, or unhappy do you feel right now? |
|  | 1. How scared, fearful, or afraid do you feel right now? |
|  | 1. How serene, content, or peaceful do you feel right now? |
|  | 1. How stressed, nervous, or overwhelmed do you feel right now? |
| **Response options** | 5-point scales: *Not at all – A little bit – Moderately – Quite a bit – Extremely* |

**Donation Intention (France et al., 2014)**

| **Instructions** | N/a |
| --- | --- |
| **Items** | 1. I plan to donate blood in the next 8 weeks. *(disagree/agree)* |
|  | 1. How likely is it that you will donate blood in the next 8 weeks? *(unlikely/likely)* |
|  | 1. I will donate blood in the next 8 weeks. *(unlikely/likely)* |
| **Response options** | 7-point scales anchored by wording as noted in item wording |

**Donation Willingness**

| **Instructions** | N/a |
| --- | --- |
| **Items** | 1. I would like to donate blood in the near future. |
|  | 1. I would be willing to donate blood if I had the opportunity in the coming weeks. |
| **Response options** | 7-point scales anchored by *not at all* and *very much so* |

**Cybersickness symptoms (selected from Simulator Sickness Questionnaire; Kennedy et al., 1993)**

| **Instructions** | *Study 1:* Indicate the degree to which the symptoms list below are affecting you right now.  *Study 2:* Indicate the degree to which you experienced the symptoms listed below during the Virtual Reality waiting/refreshment area. |
| --- | --- |
| **Items** | 1. General discomfort |
|  | 1. Eye strain |
|  | 1. Difficulty focusing |
|  | 1. Nausea |
|  | 1. Blurred vision |
|  | 1. Stomach awareness (discomfort just short of nausea) |
| **Response options** | 4-point scales: *None – Slight – Moderate – Severe* |

**Presence (Brief Presence Questionnaire v.3; Witmer, Jerome, & Singer, 2005)**

| **Instructions** | The next set of questions relate to your experiences during the Virtual Reality waiting/refreshment area. Some questions may seem less relevant to your experience - please answer according to your best judgment. |
| --- | --- |
| **Items** | 1. How much were you able to control events? |
|  | 1. How responsive was the environment to actions that you initiated (or performed)? |
|  | 1. How natural did your interactions with the environment seem? |
|  | 1. How much did the visual aspects of the environment involve you? |
|  | 1. How much did the auditory aspects of the environment involve you? |
|  | 1. How natural was the mechanism which controlled movement through the environment? |
|  | 1. How compelling was your sense of objects moving through space? |
|  | 1. How much did your experiences in the virtual environment seem consistent with your real world experiences? |
|  | 1. How well could you identify sounds? |
|  | 1. How well could you localize sounds? |
|  | 1. How compelling was your sense of moving around inside the virtual environment? |
|  | 1. How much delay did you experience between your actions and expected outcomes? |
|  | 1. How proficient in moving and interacting with the virtual environment did you feel at the end of the experience? |
|  | 1. How much did the visual display quality interfere or distract you from performing assigned tasks or required activities? |
|  | 1. How much did the control devices interfere with the performance of assigned tasks or with other activities? |
|  | 1. How well could you concentrate on the assigned tasks or required activities rather than on the mechanisms used to perform those tasks or activities? |
|  | 1. How completely were your senses engaged in this experience? |
|  | 1. Were there moments during the virtual environment experience when you felt completely focused on the task or environment? |
|  | 1. How easily did you adjust to the control devices used to interact with the virtual environment? |
| **Response options** | 7-point scales anchored by *not at all* and *very* |

**Environment-specific Immersion**

| **Instructions** | Please indicate the extent to which you agree with the following statements. |
| --- | --- |
| **Items** | 1. The virtual reality environment of the Blood Donation Centre waiting/refreshment area seemed realistic to me. *(not at all/completely)* |
|  | 1. During the virtual reality experience, it was as if I was really in a Blood Donation Centre waiting/refreshment area. *(not at all/completely)* |
|  | 1. If I were to visit a real Blood Donation Centre waiting/refreshment area, I imagine it would be similar to my experience in the virtual reality Centre. *(not at all/completely)* |
|  | 1. It was difficult for me to imagine that I was in a Blood Donation Centre waiting/refreshment area during the virtual reality experience. *(not at all/extremely)* |
|  | 1. I think that the virtual reality Blood Donation Centre waiting/refreshment area experience does not match what it is really like to be in a Centre. *(disagree completely/agree completely)* |
| **Response options** | 7-point scales anchored by wording as noted in item wording |

**Task difficulty**

***Study 2 only***

| **Instructions** | N/a |
| --- | --- |
| **Items** | *Control condition:* |
|  | 1. During the Virtual Reality waiting area, you were asked to count how many times a dot touched a rectangle. How difficult vs. easy did you find this task? |
|  | *Intervention condition:* |
|  | 1. During the Virtual Reality waiting area, you were asked to engage in a breathing task. How difficult vs. easy did you find this task? |
|  | 1. During the Virtual Reality waiting area, you were asked to think about the positive aspects of the situation. How difficult vs. easy did you find this task? |
|  | 1. During the Virtual Reality waiting area, you were asked to picture something that makes you happy. How difficult vs. easy did you find this task? |
| **Response options** | 5-point scales anchored by *not at all* and *extremely* |

**Demographics**

1. There are a variety of reasons why someone might be ineligible to donate blood (e.g., medical history, recent travel, lifestyle). Please choose the option that best describes your beliefs about your current eligibility to donate blood.
   - I believe I am currently eligible to donate blood.
   - I believe I am currently ineligible to donate blood.
   - I am unsure of my current eligibility to donate blood.
2. Which of the following is true? Consider a successful blood donation to be a donation attempt that you fully completed.
   - I have successfully made one or more blood donations in the past.
   - I have attempted or intended to make a blood donation in the past, but have never been able to successfully complete the process
   - I have not attempted to make a blood donation in the past.

*(if past successful donation/attempt)*

- 1. What blood product(s) did you donate?
     - Whole Blood
     - Plasma
     - Platelets
     - Unsure
  2. Approximately how many times have you donated blood in total?
     - 1
     - 2
     - 3
     - 4
     - 5
     - 6
     - 7
     - 8
     - More than 8
  3. Have you ever experienced an adverse event (e.g., fainting) from donating blood?
     - Yes
     - No

1. Before today, approximately how many hours have you spent using a Virtual Reality headset?
   - 1-2 hours
   - 3-4 hours
   - 5-6 hours
   - 7-8 hours
   - 9-10 hours
   - More than 10 hours
2. To what degree would you consider yourself an expert with regard to playing video games / engaging in virtual environments?
   - 0 No expertise
   - 1
   - 2
   - 3
   - 4
   - 5
   - 6 Highly expert
3. What is your age (in years)?
4. What is your gender?
   - Male
   - Female
   - Nonbinary
   - Other
5. What is your ethnicity (select all that apply)?
   - White/Caucasian
   - North East Asian (e.g. Mainland China, Hong Kong, Japan, Macau, Mongolia, Korea, Taiwan, Tibet)
   - South East Asian (e.g. Thailand, Malaysia, Vietnam, Cambodia, Singapore, Indonesia, Philippines)
   - Southern/Central Asian (e.g. India, Pakistan, Bangladesh, Sri Lanka, Nepal, Afghanistan)
   - Middle Eastern/North African
   - African
   - African-American
   - Indigenous Australian/Torres Strait Islander
   - Hawaiian/Pacific Islander
   - Hispanic/South American/Central American
   - Other (please specify)
